# ParaBTM: A Parallel Processing Framework for Biomedical Text Mining on Supercomputers

**DOI:** 10.3390/molecules23051028

**Published:** 2018-04-27

**Authors:** Yuting Xing, Chengkun Wu, Xi Yang, Wei Wang, En Zhu, Jianping Yin

**Affiliations:** 1 School of Computer Science, National University of Defense Technology, Changsha, Hunan 410073, China; xingyuting16@nudt.edu.cn (Y.X.); yangxi1016@nudt.edu.cn (X.Y.); g.webywang@gmail.com (W.W.); enzhu@nudt.edu.cn (E.Z.); 2 School of Computer Science and Network Security, Dongguan University of Technology, Dongguan, Guangdong 523808, China; jpyin@dgut.edu.cn

**Keywords:** biomedical text mining, big data, Tianhe-2, parallel computing, load balancing

## Abstract

A prevailing way of extracting valuable information from biomedical literature is to apply text mining methods on unstructured texts. However, the massive amount of literature that needs to be analyzed poses a big data challenge to the processing efficiency of text mining. In this paper, we address this challenge by introducing parallel processing on a supercomputer. We developed paraBTM, a runnable framework that enables parallel text mining on the Tianhe-2 supercomputer. It employs a low-cost yet effective load balancing strategy to maximize the efficiency of parallel processing. We evaluated the performance of paraBTM on several datasets, utilizing three types of named entity recognition tasks as demonstration. Results show that, in most cases, the processing efficiency can be greatly improved with parallel processing, and the proposed load balancing strategy is simple and effective. In addition, our framework can be readily applied to other tasks of biomedical text mining besides NER.

## 1. Introduction

With the rapid development of biotechnology, the amount of biomedical literature is growing exponentially. For instance, PubMed (https://www.ncbi.nlm.nih.gov/pubmed/), the most recognized biomedical literature database, indexes over 28 million entries for biomedical literature. Most of that information is presented in the form of unstructured texts. It is almost impossible for any domain expert to digest such a massive amount of information within a short period of time. Therefore, automated tools are essential for a systematic understanding of literature. To deal with the literature big data challenge, text mining methods are commonly applied to extract relevant knowledge from vast amounts of literature, and this has become a prominent trend in recent years [1].

Typical tasks of biomedical text mining include named entity recognition and relation extraction. One of the most fundamental tasks of biomedical text mining is named entity recognition (NER). Its task is to recognize target entities that represents key concepts from unstructured biomedical texts, such as proteins, genes, mutations, diseases, etc. There are some existing start-of-art biomedical tools that use text mining methods to identify some specific types of entities, such as mutations [2,3], genes [4,5], and diseases [6,7]. Most of these tools can achieve satisfactory recognition performance (F score over 80%) on standard datasets.

Relation extraction (RE) is a process that typically follows NER and aims to discover semantic connections between entities. Nowadays, there are a number of RE tools using different methods to identify biomedical entity interactions [8,9], such as drug–gene relationships [10,11,12], gene–disease relationships [9,13,14] and protein–protein interaction [15]. Some of them can achieve high F scores (over 80%) on several annotated datasets.

NER and RE are the preliminary steps in mining information from literature. With the uncovered facts, it is possible to construct a complex knowledge graph, which can assist new knowledge discovery and hypotheses generation. In order to achieve this goal, it is necessary to process as many articles as possible. However, text mining procedures are time consuming. BioContext, for instance, an integrated text mining system for large-scale extraction and contextualization of biomolecular events, took nearly 3 months to complete a full run of the system, which analyzed 20 million MEDLINE abstracts and several hundred thousand PMC open access full-texts using 100 concurrent processes [16]. In addition, some text mining tools, like GNormPlus [17] require a substantial amount of memory (≥5 GB), due to the necessity of loading a large gene dictionary and complementary data structures. Consequently, commodity servers cannot fulfil the computation and storage demands of large-scale text mining. Cloud-based solutions in Map-Reduce mode can partially fulfil computational resource demands. However, practically speaking, many text mining components were written in different languages, and they are dependent on a complex collection of third-party libraries, which prevents them from being readily transplanted into a high-level framework, like Hadoop and Spark. In addition, we dived into the details of load balancing, which cannot be readily supported by Map-Reduce.

An alternative solution to address this computational challenge is to harness the power of high performance computers. High performance computers (HPC) like Tianhe-2 [18] represent high-end computing infrastructures that have traditionally been used to solve compute-intensive scientific and engineering problems. The system configuration of Tianhe-2 is listed in Table 1.

Although the software stack on Tianhe-2 is designed and optimized for compute-intensive tasks, its high-performance architecture does provide the capability and capacity of big data processing. Nonetheless, to employ Tianhe-2 for big data processing is not a trivial task, which requires expert knowledge of the system architecture and parallel programming. The programming model is MPI-based (message passing interface) [18], which adds an extra dimension of complexity to normal programming languages like C/C++, Python, Java, etc. Most existing text mining tools are implemented without parallel processing. Therefore, it is necessary to develop an enabling framework that can support parallel text mining without the need to rewrite the original code. In this paper, we develop a parallel processing framework for text mining on the Tianhe-2 supercomputer. The framework integrates text mining tools as plugins. It unifies the input–output stream, implements the parallel processing across multiple compute nodes using the MPI model, and it applies a carefully crafted load balancing strategy to improve the parallelization efficiency. Without a loss of generality, we demonstrate the effectiveness of our framework using multiple NER tools as the demonstration plugins, which can recognize genes, mutations and diseases appearing in biomedical literature. More sophisticated tools of biomedical text mining can be readily integrated into the framework. In the remaining of this paper, we will introduce how paraBTM works and evaluate its performance on Tianhe-2.

## 2. Results and Discussion

To verify the effectiveness of the parallel framework, we constructed a corpus named 60K, which consists of 60,000 randomly selected articles from PubMed. For NER plugins, we chose three state-of-the-art tools (GNormPlus [17], tmVar [2], DNorm [7]), developed by NCBI (National Centre for Biotechnology Information). We measured the performance in terms of the total processing time and the average processing time (across all processes), and the total time includes the time of initialization and the actual processing time of different plugins.

The 60 K corpus is presented in the NXML format, which is a standard format provided by NCBI. Titles, abstracts, and full-texts from NXML files are extracted and re-written in the PubTator format. All input and output files processed by paraBTM should follow the PubTator format and the PubTator format starts with:

<PMID>|t|<Title of the paper>
<PMID>|a|<The rest of the paper>

The output file will be appended with annotated information like named entities followed in a tab-separated way.

**Figure 1:**
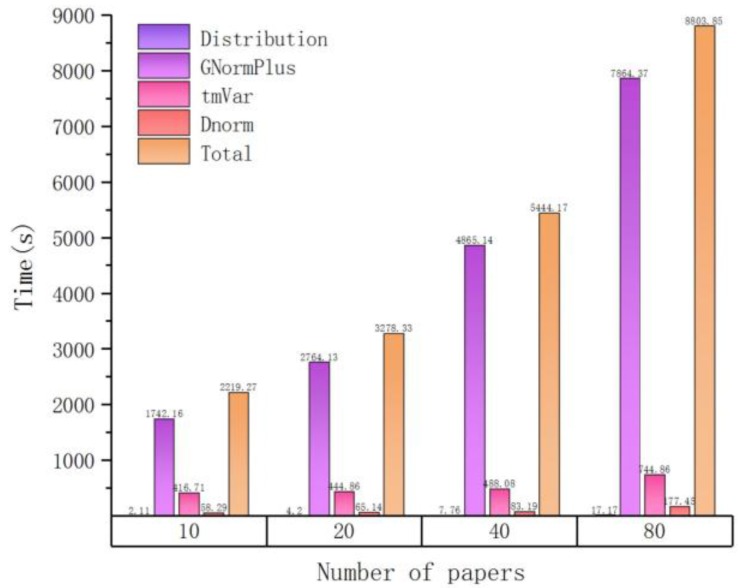
The time cost of processing different numbers of input articles in serial.

A basic fact is that the time overhead of text mining is not proportional to the number of input articles. We verified this via a single process run over several groups of randomly selected articles. The result is depicted in Figure 1. Here, different colors represent different processing plugins. Related numbers are also listed in Table 2.

**Figure 2:**
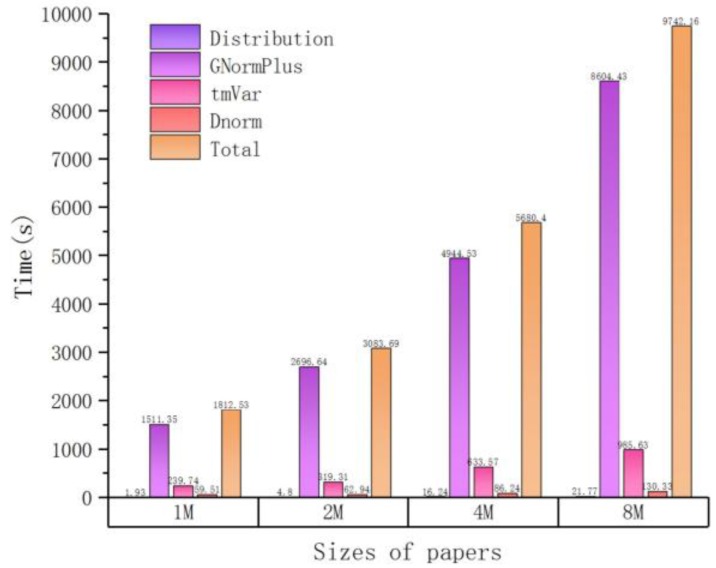
The time cost of processing different input sizes in serial.

As the number of input files increases, the time cost also increases but not linearly. For example, when the number of input articles is equal to 10, it takes about 36 min for tagging entities, and when the number of articles increases to 100, the spent time is about 3 h (180 min). This can be attributed to another important observation, that is, the total processing time is approximately proportional to the total input size (sum of file lengths as measured by number of characters), which is illustrated in Figure 2 (size unit is MB, mega-bytes) and Table 3. The workload of each plugin can be better estimated by the total length of input files, which is the basis for our load balancing strategy in the following part.

**Figure 3:**
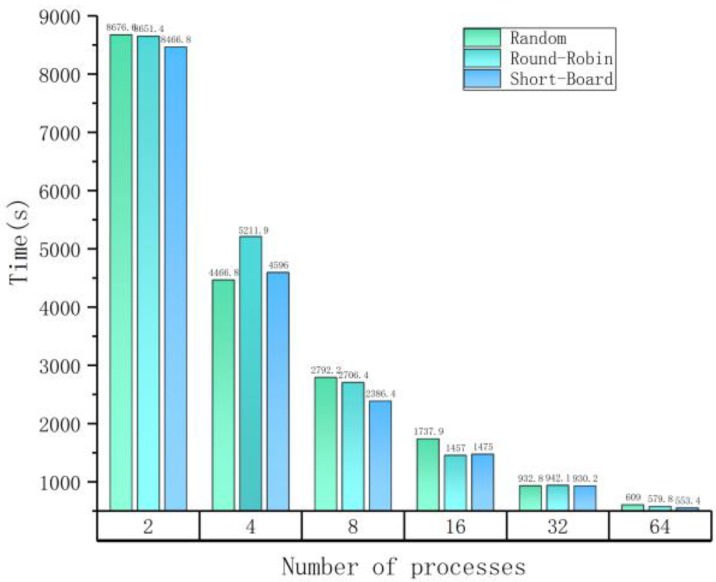
Effects of different load balancing strategies.

Figure 3 shows the time spent on paraBTM processing with different numbers of parallel processes on an input dataset of 16 MBs (including 175 articles) which is composed of articles randomly selected from the 60 K corpus. Parallel processing greatly reduces the processing time and different load-balancing strategies do affect the parallel efficiency. paraBTM costs about 500 s (under the Short-Board balancing strategy) when 64 processes are employed, which is around 1/16 the processing time of 2 processes. To note, each process needs to carry out initialization for every plugin, which means you cannot reduce the total processing time any further if the initialization time cost becomes the majority part.

To profile different load balancing strategies, we summarize their effects under different parallel scales, as listed in Table 4. In all 6 test cases, the Short-Board strategy is the best in 4 cases and 2nd best in 2 remaining cases. We employ the load balancing efficiency (LBE) to quantify the effects of different strategies. Here, LBE is defined as:

LBE = AET/MET(1)

Here, AET is the average execution time and MET is the maximum execution time. According to the above definition, the maxima of LBE is 1 (achieved if AET is equal to MET) and a greater LBE represents a better load balancing efficiency.

**Figure 4:**
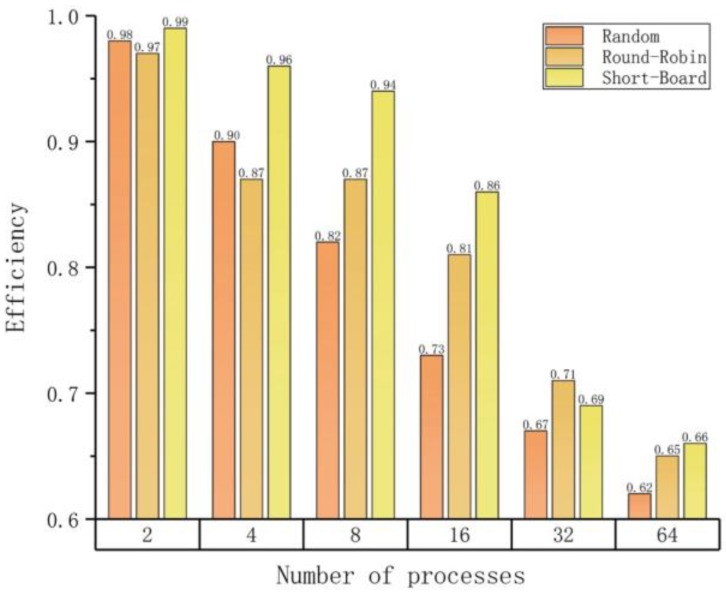
Load balancing efficiencies.

Figure 4 shows that the Short-Board strategy exhibits the best LBE in almost all test cases. However, LBE values drop significantly when the number of parallel processes is greater than 16 in the 16M test set. The reason is that this test set contains only 175 articles, which means each process will only process two articles on average. If the input data set is big enough, the LBE will be maintained at a satisfactory level.

We also conducted an experiment on the whole 60 K corpus (61,078 articles). Table 5 shows that it took over 12 h to process 61,078 papers through three NER plugins (128 nodes under the Short-Board strategy, each node runs 5 processes). According to the results, we can see that parallelization greatly enhances the processing efficiency. To note, the speed-ups of different plugins differ as each plugin has its own characteristic computation and memory access patterns. To carry out a full-scale processing on the whole PMC-OA full-text dataset (over 1 million), it will take about 200 h if we only use 128 nodes. Fortunately, the computation capacity of Tianhe-2 is enormous, and we can reduce the total time down to several hours by harnessing the power of a few thousand nodes (over 16,000 available on Tianhe-2). We plan to carry out a full analysis on the whole PubMed dataset (the real large-scale biomedical texts) in the future. However, the cost of such a full run is currently beyond our funding support. We are currently in the application process of a bigger grant for this large-scale analysis. In our previous study, we have demonstrated that using text mining on a larger dataset does provide more comprehensive and insightful results compared with using a small dataset (say, can be handled by a few people) using thyroid cancer as a case study [19].

## 3. Materials and Methods

### 3.1. Data Sources and Storage

The biomedical literature has typical characteristics of large quantity, professional content, public resources, easy-accessibility, etc. Because of these characteristics, biomedical literature data has become one of the most noticeable data in biomedical field. For example, PubMed Central (PMC) is a free digital repository that archives publicly accessible articles. Until now, PMC has contained over 4.1 million references to full-text journal papers, covering a wide range of biomedical fields, and the literature data is stored in NXML format, from which we can extract some parts according to our interest.

However, most of the state-of-art NER tools do not support parallel processing, and it would take an enormous amount of time if we want to process the massive set of biomedical literature. One feasible solution is to harness the computing power provided by HPC systems by implementing a parallel NER processing framework. With this framework, text mining tools can be easily integrated into the framework and developers will not need to consider the details of parallel processing.

**Figure 5:**
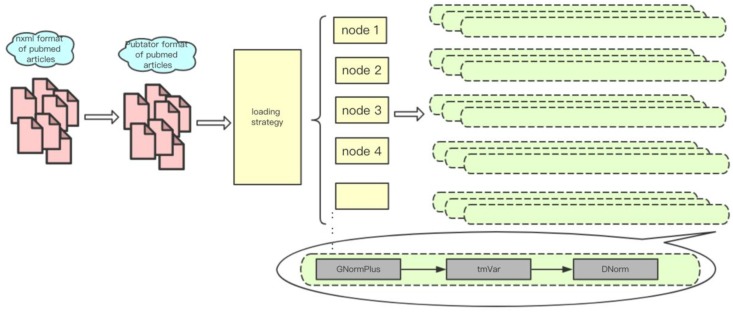
The implementation and deployment of paraBTM in large-scale parallel environment.

There are different levels of parallelism in text mining tasks. First, each input article is relatively independent; secondly, multiple sentences in each of the articles can be approximately regarded as independent. However, in practice, we usually use a single file as a processing unit, the reason is that many text mining tools spend a substantial amount of time to initialize on each processing pass. In addition, the memory size also limits the number of processes that can run in parallel on each computing node. For instance, on Tianhe-2 each node is equipped with 24 cores and 64 GB of memory, and the stable memory that users can control is about 50 GB (the operating system and other necessary tools need to use about 10 GB). The memory costs of a typical TmVar and gnormplus run for NER can be up to 5 GB and 10 GB. Therefore, at most 5 GNormPlus processes and 10 TmVar processes can run on one node. Figure 5 shows the implementation and deployment of a text mining system (paraBTM) in large-scale parallel environment.

### 3.2. Parallel Processing

#### 3.2.1. MPI-Based Multi-Node Computation

The message passing interface (MPI) is a standard model for parallel programming on HPCs. It is well established over 20 years, and has been implemented in different sorts of programming languages including C/C++ and Python. Our method can run on any supercomputer or cluster configured with MPI support. To note, different supercomputers might have different node configurations. When running on other platforms, the configuration (RAM, number of concurrent processes) might have to change accordingly.

In this work, we use MPI4PY (http://mpi4py.scipy.org/docs/) to implement the parallel processing. MPI4PY is a well-regarded, clean, and efficient implementation of MPI for Python. Our framework can simultaneously submit many jobs to cores distributed across computing nodes in Tianhe-2.

#### 3.2.2. Load Balancing Strategy

A typical challenge in parallel computation is load unbalance, that is, workload is unevenly distributed among nodes, making some nodes very busy for a long time and others idle [20]. In this paper, we address this problem by designing an effective load balancing strategy.

Given a set F of files to be processed $F_{0},F_{1},F_{2}\ldots F_{N-1},F_{n}$, we initialize processes $P_{0},P_{1},P_{2},\ldots \ldots ,P_{n-1},P_{n}$, and the number of processes is N, the problem is to allocate each file $F_{x}$ to an appropriate process $P_{rank}$.

**Figure 6:**
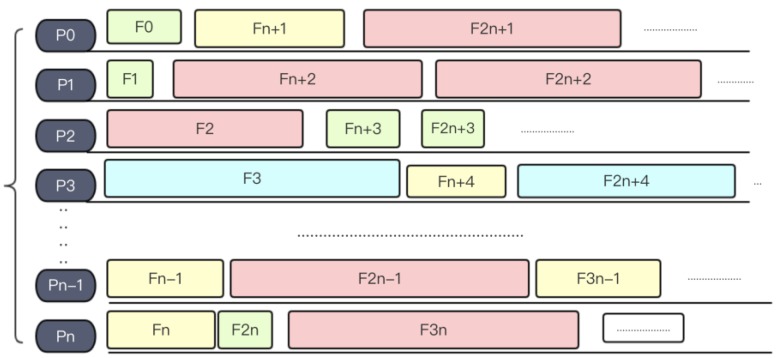
Random load balancing strategy.

A naive solution is to randomly distribute target files into nodes. We can simply distribute files to by modulo operation rank=mod(P,size), *P* and size represent the position of the target file in the file list and number of processes respectively, and file $F_{p}$ finally should be sent to $P_{rank}$. According to the formula, each file F  to be processed is distributed to process rank=P % size in turn. As the files are arranged in a random order, this process is actually a simulation of random distribution. This is a naïve strategy and easy to implement. However, this strategy does not take into consideration the length of each file, and will very likely cause an unbalanced load distribution, which would detriment the overall parallel efficiency. For instance, if the total length of files assigned to one specific node is far larger than others, then the overall running time will be prolonged until this slowest node finishes. Figure 6 shows an example of the naïve random load balancing strategy.

**Figure 7:**
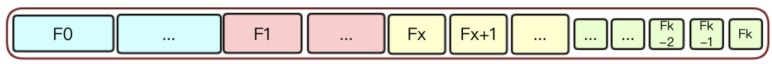
A sorted file list.

**Figure 8:**
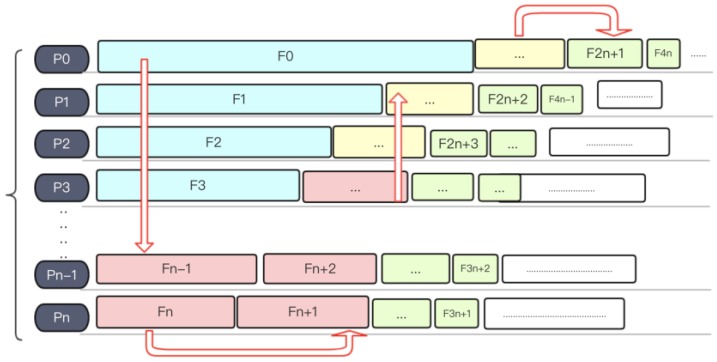
Round-robin algorithm.

A slightly more complex load balancing method is the round-robin (RR) method. Round-robin algorithm is a term that originally comes from the field of operating systems. Here, the general idea inspires us to mix small files with large files together into one process. After sorting files by size (see Figure 7), the system will assign files into processes in a snakelike way, making the size of files loaded in every process remains relatively balanced. Figure 8 shows an example of RR algorithm.

The round-robin method also allocates the same number of files, and its serpentine way of load assignment ensures that the total size of the files in each process remains relatively balanced, since files were sorted by size in advance. However, in some circumstances, the lengths of input articles can be very biased, say, some files are extremely long while many others are short. In such cases, the RR method fails.

**Figure 9:**
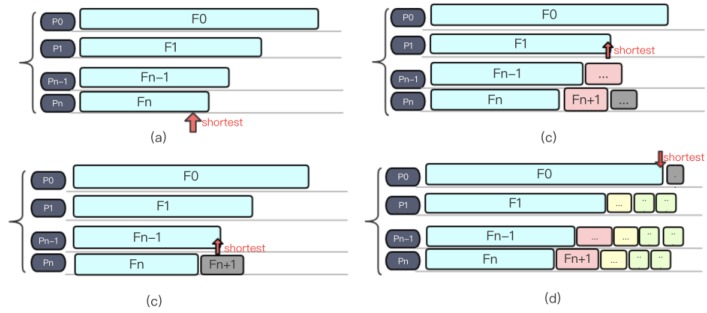
A demonstration of the Short-Board load balancing algorithm.

**Figure 10:**
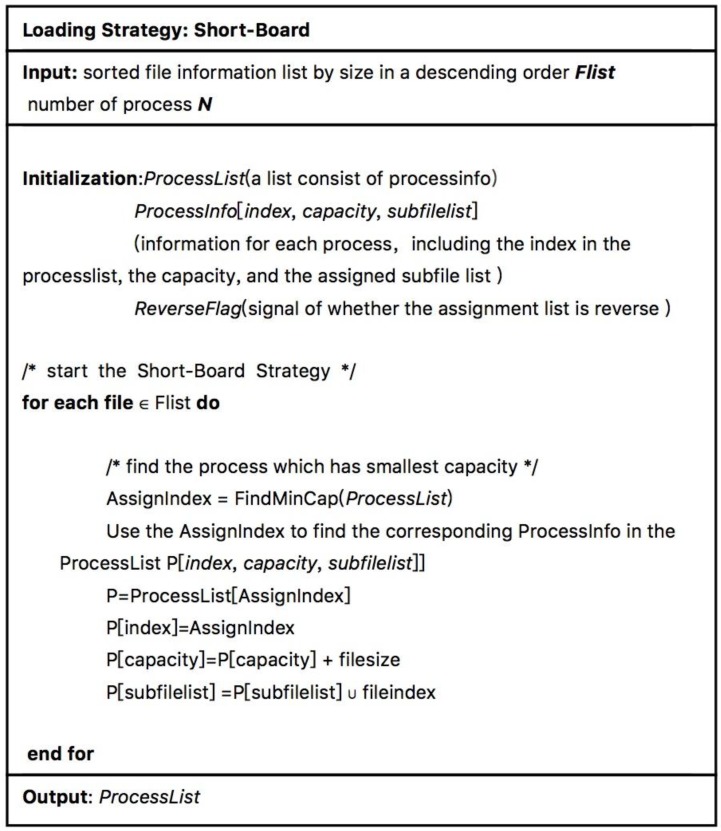
The pseudo code of Short-Board.

Instead of assignments based on the number of files, we proposed our “Short-Board” method. Firstly, the files that need to be processed are sorted in descending order according to the length of each file, and then files that need to be processed in the file list are sequentially fetched out and dispatched to the process whose current load is the smallest. Figure 9a–d shows an example of Short-Board algorithm. The pseudo code of Short-Board is shown in Figure 10.

## 4. Conclusions

In this paper, we present paraBTM, a parallel framework for biomedical text mining developed on the Tianhe-2 supercomputer. It supports different types of components as plugins and its usage is straightforward. The parallel efficiency is guaranteed by a carefully devised load balancing strategy. We evaluated the performance of paraBTM on both small- and large-scale datasets. Experimental results validate that paraBTM effectively improve the processing speed of biomedical named entity recognition. On large scale of datasets, ParaBTM managed to process 60178 PubMed full-text articles in about 12 h. paraBTM is open-source and available at https://github.com/biotm/paraBTM.

## Figures and Tables

**Table 1:** The system configuration of Tianhe-2.

Items	Content
Manufacturer	NUDT
Cores	3,120,000
Memory	1,024,000 GB
CPU	Intel Xeon E5-2692v2 12 C 2.2 GHz
Interconnect	TH Express-2
Linpack Performance(Rmax)	33,862.7 TFlop/s
Theoretical Peak(Rpeak)	54,902.4 TFlop/s
HPCG [TFlop/s]	580.109
Operating System	Kylin Linux
MPI	MPICH2 with a customized GLEX channel

**Table 2:** Time distribution of processing different numbers of input articles in serial.

Number of Papers	Time(s)				
	Distribution	GNormPlus	tmVar	Dnorm	Total
10	2.11	1742.16	416.71	58.29	2219.27
20	4.2	2764.13	444.86	65.14	3278.33
40	7.76	4865.14	488.08	83.19	5444.17
80	17.17	7864.37	744.86	177.45	8803.85

**Table 3:** Time cost distribution of processing different input sizes in serial.

Sizes of Papers (M)	Time (s)				
	Distribution	GNormPlus	tmVar	Dnorm	Total
1	1.93	1511.35	239.74	59.51	1812.53
2	4.80	2696.64	319.31	62.94	3083.69
4	16.24	4944.35	633.57	86.24	5680.40
8	21.77	8604.43	985.63	130.33	9742.16

**Table 4:** Profiling of different load balancing strategies.

(a) Maximum times on different numbers of parallel processes with different strategies.			
**Number of Processes**	**Maximum Time among All Processes (s)**		
	**Random**	**Round-Robin**	**Short-Board**
2	8676.60	8651.42	8466.89
4	4466.81	5211.96	4596.09
8	2792.29	2706.41	2386.45
16	1737.94	1457.01	1475.07
32	932.85	942.10	930.25
64	609.06	579.8	553.42
(b) Average times on different numbers of parallel processes with different strategies.			
**Number of Processes**	**Average Time of All Processes (s)**		
	**Random**	**Round-Robin**	**Short-Board**
2	8513.10	8374.08	8392.46
4	4001.38	4535.42	4393.07
8	2287.19	2354.14	2242.42
16	1265.46	1174.12	1270.33
32	624.73	666.91	640.63
64	379.08	376.06	366.84
(c) Load balancing efficiencies on different numbers of parallel processes with different strategies.			
**Number of Processes**	**Efficiency (Average/Maximum)**		
	**Random**	**Round-Robin**	**Short-Board**
2	0.98	0.97	0.99
4	0.90	0.87	0.96
8	0.82	0.87	0.94
16	0.73	0.81	0.86
32	0.67	0.71	0.69
64	0.62	0.65	0.66

**Table 5:** The processing time of 61,078 papers running on 128 processes.

Number of Processes	Time (s)				
	Distribution	GNormPlus	tmVar2.0	Dnorm	Total
1	18,934.18	5,874,482.04	654,145.38	82,455.3	6,630,016.9
128	3643	23,733	16,214	233	43,823
Speed-up (x)	5.20	247.52	40.34	353.89	151.29
